# Coordinated features in jaw and neck muscle activities induced by chewing of soft and hard gum in healthy subjects

**DOI:** 10.1002/cre2.413

**Published:** 2021-03-09

**Authors:** Tomohiro Ishii, Noriyuki Narita, Hiroshi Endo, Masanobu Wakami, Masakazu Okubo, Takeshi Uchida, Ikuo Kantake, Koh Shibutani

**Affiliations:** ^1^ Department of Removable Prosthodontics Nihon University School of Dentistry at Matsudo Chiba Japan; ^2^ Research Institute of Oral Science Nihon University School of Dentistry at Matsudo Chiba Japan; ^3^ Human Technology Research Institute National Institute of Advanced Industrial Science and Technology (AIST) Ibaraki Japan; ^4^ Department of Oral Health Science Division of Oral Function and Rehabilitation Nihon University School of Dentistry at Matsudo Chiba Japan; ^5^ Dental Support Co. Ltd. Chiba Japan; ^6^ Department of Anesthesiology Nihon University School of Dentistry at Matsudo Chiba Japan

**Keywords:** chewing gum, masticatory muscles, masticatory systems, neck muscles, surface electromyography

## Abstract

**Backgrounds:**

Jaw and neck muscles may be activated by chewing load using a hard food. However, it remains unclear how effects the gum hardness to the coordinated features in jaw and neck muscle activities during chewing performance.

**Objectives:**

This study was conducted to quantitatively elucidate the effects of the hardness of the gum on coordinated features in jaw and neck muscle activities using intermuscular EMG–EMG transfer function and EMG–EMG coherence function analyses in 18 healthy subjects.

**Methods:**

Jaw and neck muscle activities were aggregated into the first peak frequency of the power spectrum, and power, gain, phase, and coherence parameters between jaw and neck muscle activities were examined in the first peak frequencies during soft and hard gum chewing.

**Results:**

The first peak frequency was not significantly different between soft and hard gum chewing. In contrast, power values of the jaw and neck muscles were significantly increased by chewing of hard gum as compared with soft gum, whereas gain, phase, and coherence were not significantly changed by gum hardness.

**Conclusions:**

The chewing rhythm, the quantitative and temporal coordination, and the functional coordination in jaw and neck muscle activities were not changed during soft and hard gum chewing, as well as increased jaw and neck muscles activities. It is therefore concluded that the chewing rhythmicity and jaw and neck muscles coordination accompanied with the increased jaw and neck muscle activities are maintained under the condition of the chewing load using gum hardness in the healthy individuals.

## 1 INTRODUCTION

Physiologically coordinated jaw and neck muscle activities has been evaluated in regard to jaw clenching (Giannakopoulos, Hellmann, et al., 2013; Giannakopoulos, Schindler, et al., 2013; Kibana et al., 2002; Politti et al., 2010), jaw opening and closing movements (Eriksson et al., 1998, 2000), and chewing (Guo et al., 2017; Häggman‐Henrikson et al., 2013; Häggman‐Henrikson & Eriksson, 2004; Igarashi et al., 2000; Shimazaki et al., 2006). For example, bilateral jaw clenching was shown to produce bilateral neck muscle activation and unilateral jaw clenching side produce predominant neck muscle activity (Kibana et al., 2002). In addition, neck muscle activity was found to occur concomitantly with maximal jaw‐opening and jaw‐closing cycles in parallel with head extension‐flexion movements (Eriksson et al., 1998, 2000), while other results indicated that neck muscles, especially the sternocleidomastoid, may be activated in response to chewing load related to food size and food hardness (Häggman‐Henrikson et al., 2013). In consideration of these previous findings, jaw and neck muscles may be activated by chewing load with hard food bolus. However, it remains unclear how chewing a hard gum affects to the coordinated features in jaw and neck muscle activities in healthy subjects.

Recently, the coordinated features in jaw and neck muscle activities during rhythmical chewing were examined in a quantitative manner using intermuscular EMG–EMG transfer function and EMG–EMG coherence function analyzes in healthy subjects (Ishii et al., 2016). Results of that study suggested that the first peak frequency, resulted in the power spectrum of chewing‐related jaw and neck muscle activities, may fit with chewing rhythmicity. Furthermore, the predominantly coordinated features in jaw and neck muscle activities were presented in chewing side, while the non‐chewing side neck muscle presented the irregular temporality between jaw and neck muscle activities in the rhythmical chewing. From these, it is considered that intermuscular EMG–EMG transfer function and EMG–EMG coherence function analyzes may present the physiologically coordinated features in chewing‐related jaw and neck muscle activities induced by chewing load using gum hardness in healthy subjects. This study is conducted to clarify the effects of gum hardness on physiological features in chewing rhythmicity, the quantitative and temporal coordination and the functional coordination between jaw and neck muscle activities during chewing performance in healthy subjects.

The aim of this study was to investigate the effects of the gum hardness on coordinated features in jaw and neck muscle activities using intermuscular EMG–EMG transfer function and EMG–EMG coherence function analyzes during chewing performance.

## 2 MATERIALS AND METHODS

### 2.1 Subjects

Eighteen healthy voluntary subjects (15 males, 3 females; mean age 24.3 years, range 20–32 years) were recruited from staff members and dental students at Nihon University School of Dentistry at Matsudo. All enrolled subjects had complete dentitions. A set of standardized and validated self‐reporting questionnaires, as well as a diagnostic criteria for TMD (DC/TMD) (Schiffman et al., 2014) examination protocol for clinical characterization were used in TMD specialist. The subjects were free from pain and dysfunctions in the oromandibular, maxillofacial, head, and neck and shoulder regions. The sample size of 18 was determined using the G*Power 3 software package (noncommercial program downloaded from University of Dusseldorf, Germany) (Faul et al., 2007). Prior to the start of the study, each participants provided informed consent according to the World Medical Association's Declaration of Helsinki. This study was approved by the committee on ethics of Nihon University School of Dentistry at Matsudo.

### 2.2 Experimental procedures

The subjects were comfortably seated in an upright position without back support or a headrest. Occlusal conditions, bite force, and occlusal contact area were examined at the beginning. The subjects were also instructed to chew soft and hard gum until it reached a homogeneous condition preceding the chewing experiments.

### 2.3 Recording of occlusal conditions

Occlusal conditions of bite force and occlusal contact area were measured using 97‐μm thick pressure sensitive sheets (Dental Prescale 50H R‐type, Fuji Film Co., Tokyo, Japan) during maximal jaw clenching in the intercuspal position, and then occlusal data were scanned by occlusal diagnostic device (Occluzer FPD703, Fuji Film Co., Tokyo, Japan) for the evaluations of occlusal contact area (mm^2^), occlusal force (N), asymmetrical index (AI) for occlusal contact area (%), occlusal force (%), average pressure (MPa), and maximum pressure (MPa).

### 2.4 Recording of muscles EMG

EMG signals in the jaw and neck muscles were recorded using surface EMG electrodes, with a method described in a previous study (Ishii et al., 2016). Following skin cleaning with ethanol (76.9%–81.4%), a pair of bipolar Ag/AgCl electrodes 7 mm in diameter was attached to the skin overlying the examined muscles parallel to the direction of the muscle fibers with an interelectrode distance of 20 mm, while a ground electrode was also attached to the left ear lobe. Electrodes were positioned bilaterally on the masseter (Mm, jaw‐closing muscle), anterior temporal (Ta, jaw‐closing muscle), anterior digastric (AD, jaw opening muscle), and sternocleidomastoid (SCM, neck extensor/protrusion/rotator muscle) muscles. EMG signals were amplified (POLYGRAPH BIOELECTRIC AMPL 1253A, San‐ei MED, Tokyo, Japan), with the high frequency cut‐off filtered at 1 kHz and a time constant of 0.03 s, then digitized with 16‐bit resolution by an A/D converter (APA16‐32/2[OB] F, CONTEC, Tokyo, Japan). Obtained data were downloaded to a personal computer (IBM Think Centre, Tokyo, Japan) at a sampling rate of 1 kHz.

### 2.5 Test food and chewing performance

**FIGURE 1:**
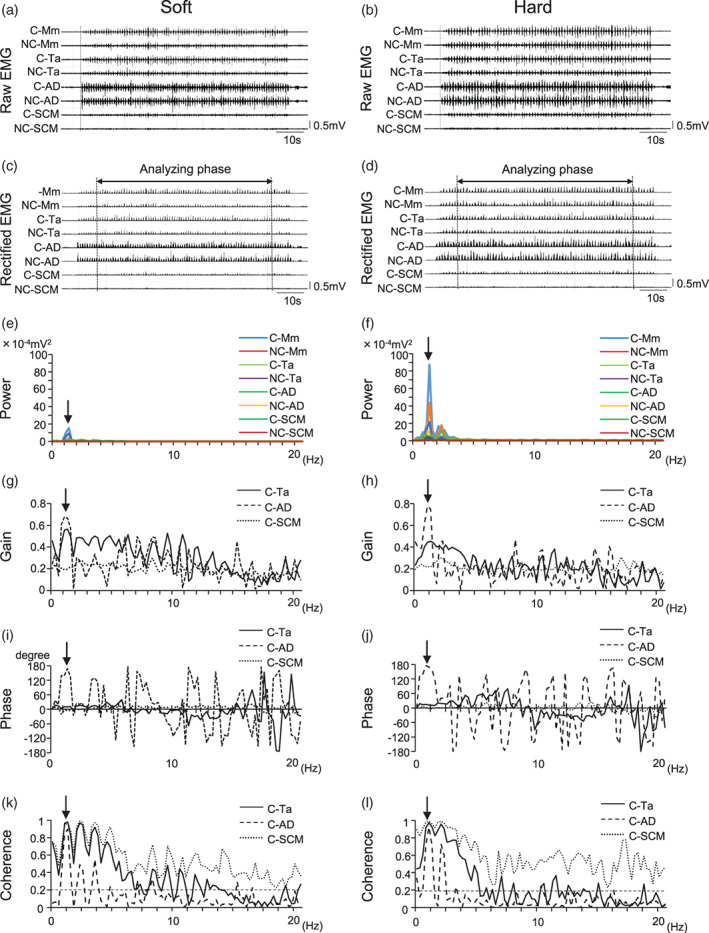
Raw and rectified EMG activities and power spectrum, as well as representative power phase and coherence in jaw and neck muscle activities with soft and hard gum chewing. Raw EMG activities (a, b) and full wave rectification of muscle activity (c, d) in jaw and neck muscles during chewing of soft (a, c) and hard (b, d) gum for 80s in a representative subject. Area between vertical lines indicates analyzing phase (61.5 s). The power spectrum for jaw and neck muscle activities by FFT from 0 to 20 Hz (e, f), and arrows indicate the first peak frequency in the power spectrum. Gain (g, h) and phase (i, j) were analyzed using transfer function analysis, while coherence (k, l) was analyzed using coherence function analysis. The 95% confidence level (0.19) for the coherence spectrum shown in (k) and (l) is presented as a horizontal line. C and NC represent chewing side and non‐chewing side, respectively. Mm, ta, AD, and SCM represent masseter muscle, anterior temporal muscle, anterior digastric muscle, and sternocleidomastoid muscle, respectively

Pieces of chewing gum differing in hardness (soft: 5.6 × 10^4^ Pa · s, hard: 9.3 × 10^6^ Pa · s tasteless gum, Lotte, Tokyo, Japan), but with identical size and shape (15 × 50 × 1 mm, 2 g) were used as the test food. Four chewing sessions were performed with these two types of gum hardness (soft and hard) on both the right and left sides in random order. First, the chewing gum was placed in the mouth at rest, then the subject was instructed to chew for 80s (Figure 1a,b). The start and stop of the chewing task were instructed by verbal commands.

### 2.6 Data analysis of muscles EMG

Muscle EMG activities were analyzed using a software package (Multi Scope EMG/Ver1.8.4, Medical Try System, Tokyo, Japan). To investigate the power spectrum during chewing, the full wave rectified EMG activity (Figure 1c,d) was used according to the methods described by previous study (Ishii et al., 2016; Nielsen et al., 1994). In these studies, rectified EMG signals are used to obtain a peak power spectrum of muscle activity that corresponds to the stride‐cycle frequency during walking (Nielsen et al., 1994) and masticatory rhythms during chewing (Ishii et al., 2016) (Figure 1e,f).

Rectified EMG signals were evaluated based on the input and output relationship of first peak signals with the transfer function which could be associated with the chewing rhythms. Because an agonist muscle activity (i.e., chewing side masseter Mm) plays an agonist role and may also receive a profound effect from the central pattern generation in the brainstem (Nakamura & Katakura, 1995). Hereafter, the letter ‘C’ is used as an abbreviation to indicate chewing side and ‘NC’ for non‐chewing side, for example, chewing and non‐chewing side Mm values are expressed as C‐Mm and NC‐Mm, respectively. C‐Mm activity was used as the input signal, while synergist (NC‐Mm, C/NC‐Ta), antagonist (C/NC‐AD), and neck (C/NC‐SCM) muscles were used as output signals. Relative strength was calculated as the gain value in transfer function analysis, which has been shown to reflect the change in variability of output signals in response to changes in input signal (Ehara et al., 2012; Peterka et al., 2006; Zhang et al., 1998). The time difference is also shown as the phase value in transfer function analysis, which represents the temporal relationship between input and output signals (Ehara et al., 2012; Sakagami et al., 2011; Zhang et al., 1998). In order to secure an adequate frequency resolution for chewing rhythm cycle and confidence value for coherence function analysis, the analysis time was determined to be 61.5 s, as previously described in detail (Ishii et al., 2016).

Transfer function *H* (*f*) between the input and output signals was also calculated using a cross‐spectral technique (Carter et al., 1973), as noted below, with *Sxx* (*f*) representing the autocorrelation function of input signals, and *Sxy* (*f*) the cross‐correlation function between input and output signals.

(1) $$H\left(f\right)=\frac{S_{\mathrm{xy}}\left(f\right)}{\mathrm{Sxx}\left(f\right)}$$

Gain |*H* (*f*)| and phase Φ (*f)* were calculated as follows, with *H*_R_ (*f)* the real part of *H* (*f)* and *H*_*I*_ (*f)* the speculated part of *H* (*f*).

(2) $$\left|H\;\left(f\right)\right|=\frac{\left[H_{R}\;{\left(f\right)}^{2}+H_{I}\;{\left(f\right)}^{2}\right]}{\mathrm{Sxx}\left(f\right)}$$

(3) $$\emptyset \left(f\right)=\tan^{-1}\left[\frac{H_{I}\;\left(f\right)}{H_{R}\;\left(f\right)}\right]$$

Coherence (Coh^2^ (*f*)) was obtained as shown below, with *Sxx* (*f)* and *Syy* (*f)* representing the autocorrelation function of signals. Coherence, as functional coordination (Laine & Valero‐Cuevas, 2017) indicates synchrony between two signals using values from 0 to 1.

(4) $${\mathrm{Coh}}^{2}\;\left(f\right)=\frac{{\left|\mathrm{Sxy}\;\left(f\right)\right|}^{2}}{\left[\mathrm{Sxx}\;\left(f\right)\mathrm{Syy}\;\left(f\right)\right]}$$

FFT results of jaw and neck muscle activities showed the appearances of the high amplitude in the first peak and low amplitudes in integer multiples at a high frequency (Figure 1e,f), and integer multiple second, third, fourth, and fifth peaks are considered as harmonics (Smith 1997). Therefore, gain, phase by transfer function analysis and coherence by coherence function analysis in the present study were analyzed for the first peak frequency of the power spectrum in jaw and neck muscle activities during rhythmical chewing.

### 2.7 Analysis items of muscles EMG

We obtained power values on the first peak frequency of the power spectrum for jaw closing (C‐/NC‐Mm, C‐/NC‐Ta), jaw opening (C‐/NC‐AD), and neck (C‐/NC‐SCM) muscle activities during right and left side chewing with soft and hard to evaluate the effect of gum hardness on power values. Gain, phase, and coherence in the first peak frequency of the power spectrum of jaw and neck muscle activities were also analyzed for NC‐Mm, C‐/NC‐Ta, C‐/NC‐AD, and C‐/NC‐SCM. C‐Mm findings of a representative subject are shown in Figure 1g–l. In addition, phase and coherence for the first peak frequency of the power spectrum of jaw and neck muscle activities were also compared between soft and hard gum during rhythmical chewing.

### 2.8 Statistical analysis of muscles EMG

The assumption of normality of the data obtained for the first peak frequency of jaw and neck muscle activities, and the power, gain, phase, and coherence in the first peak frequency were tested by Kolmogorov–Smirnov test. Paired *t*‐test or Wilcoxon signed‐ranks test were used to compare the data of the examined muscles (C‐Mm and/or NC‐Mm, C‐/NC‐Ta, C‐/NC‐AD, C‐/NC‐SCM) between soft and hard gum (Häggman‐Henrikson et al., 2013; Iguchi et al., 2015; Takada et al., 1994). All statistical analyzes were performed using SigmaStat, v. 4.0 (Systat Software, Inc, CA, USA) and results were considered to be significant when the value for comparison was less than 5%.

## 3 RESULTS

### 3.1 Occlusal conditions

Occlusal contact area, occlusal force, averaged pressure, maximum pressure, and occlusal contact area and occlusal force AI values for the 18 healthy subjects were calculated. The average and standard deviation of area and area AI were 27.6 ± 12.3 mm^2^ and 17.3 ± 16.0%, respectively, while the average and standard deviation of force and force AI were 964.8 ± 308.7 N and 16.8 ± 16.5%, respectively, and the average and standard deviation of average pressure and maximum pressure were 42.7 ± 7.9 and 103.6 ± 14.4 MPa, respectively.

### 3.2 First peak frequency

In each of the subjects, the frequency as the first peak of the power spectrum were consistent across all of muscle for jaw closing (C‐/NC‐Mm, C‐/NC‐Ta), jaw opening (C‐/NC‐AD), and neck (C‐/NC‐SCM) muscle activity within the subject during right and left side chewing with soft and hard gum. The average and standard deviation for first peak frequency were 1.15 ± 0.16 Hz for right side and 1.15 ± 0.16 Hz for left side chewing of soft gum, and 1.17 ± 0.16 and 1.16 ± 0.16 Hz, respectively, for hard gum, with no significant difference seen between the first peak frequencies for soft and hard gum chewing on the right and left sides (Wilcoxon signed‐rank test).

### 3.3 Power

Power for the first frequency of jaw closing (C‐/NC‐Mm, C‐/NC‐Ta), jaw opening (C‐/NC‐AD), and neck (C‐/NC‐SCM) muscle activities was evaluated during right and left side chewing with soft and hard gum (Table 1). The power of the jaw‐closing muscles (C‐/NC‐Mm, C‐/NC‐Ta) was significantly increased during left and right side chewing with hard as compared to soft gum (Wilcoxon signed‐rank test), *p* < .01) (Table 1). The power of the jaw opening muscles (C‐/NC‐AD) was significantly increased during left and right side chewing with hard as compared to soft gum (Wilcoxon signed‐rank test, *p* < .01, paired *t* test, *p* < .05) (Table 1). The power of the neck muscles (C‐/NC‐SCM) was significantly increased during left and right side chewing with hard as compared to soft gum (Wilcoxon signed‐rank test), *p* < 0.01) (Table 1).

**TABLE 1:** Power values for jaw and neck muscle activities during soft and hard gum chewing

	Left side chewing		Right side chewing	
	Soft (mean ± SD) (×10^−4^ mV^2^)	Hard (mean ± SD) (×10^−4^ mV^2^)	Soft (mean ± SD) (×10^−4^ mV^2^)	Hard (mean ± SD) (×10^−4^ mV^2^)
C‐Mm	28.10 ± 25.10	53.00 ± 41.60***	37.80 ± 36.60	82.40 ± 68.40***
NC‐Mm	12.00 ± 17.90	22.10 ± 22.40***	12.20 ± 14.10	38.60 ± 26.60***
C‐Ta	20.40 ± 15.50	43.80 ± 42.60***	13.60 ± 15.70	45.50 ± 53.40***
NC‐Ta	8.00 ± 10.50	17.10 ± 18.20***	9.45 ± 5.84	16.82 ± 14.57**
C‐AD	1.60 ± 1.83	2.74 ± 2.67***	1.89 ± 2.73	2.66 ± 2.60^*^
NC‐AD	3.40 ± 3.50	5.80 ± 5.30***	3.51 ± 5.97	5.36 ± 6.08***
C‐SCM	0.06 ± 0.08	0.18 ± 0.28***	0.08 ± 0.12	0.19 ± 0.34***
NC‐SCM	0.01 ± 0.01	0.02 ± 0.02***	0.03 ± 0.03	0.11 ± 0.14***

*Note*: The power values for jaw and neck muscle activity (NC‐Mm, C‐/NC‐Ta, C‐/NC‐AD, C‐/NC‐SCM) during hard gum chewing were significantly higher than during soft gum chewing for both right and left side chewing (Wilcoxon signed‐rank test).

Abbreviations: AD, anterior digastric muscle; C, chewing side; Mm, masseter muscle; NC, non‐chewing side; SCM, sternocleidomastoid muscle; SD, standard deviation; Ta, anterior temporal muscle.

* *p* < .05, paired *t* test.

** *p* < .05.

*** *p* < .01.

### 3.4 Gain

**FIGURE 2:**
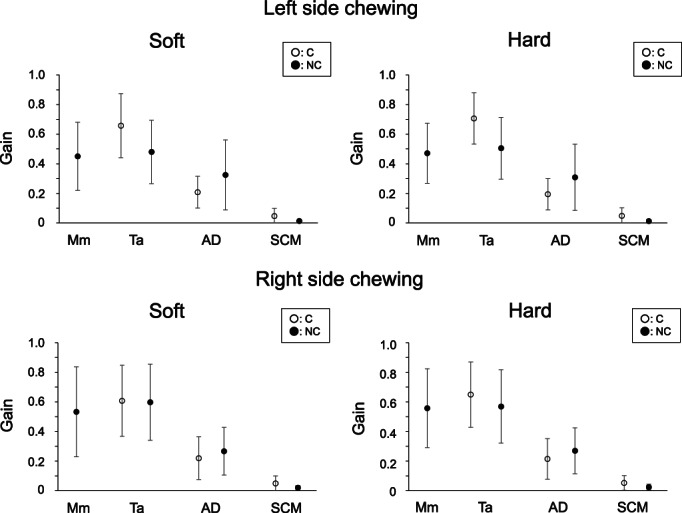
Gain for jaw and neck muscle activities. Mean and standard deviation of gain values for the first peak frequency of jaw closing (NC‐mm, C‐/NC‐ta), jaw opening (C‐/NC‐AD), and neck (C‐/NC‐SCM) muscle activities are shown. No significant differences were seen for gain between soft and hard gum chewing (Wilcoxon signed‐rank test, paired *t*‐test). The prefixes of ‘C’ (open circle) and ‘NC’ (black circle) denote the chewing side and non‐chewing side muscles, respectively. Mm, ta, AD, and SCM represent masseter muscle, anterior temporal muscle, anterior digastric muscle, and sternocleidomastoid muscle, respectively

Gain for the first peak frequency of jaw closing (NC‐Mm, C‐/NC‐Ta), jaw opening (C‐/NC‐AD), and neck (C‐/NC‐SCM) muscle activities was evaluated during right and left side chewing with soft and hard gum (Figure 2). No significant differences was seen for gain of jaw closing (NC‐Mm, C‐/NC‐Ta), jaw opening (C‐/NC‐AD), and neck (C‐/NC‐SCM) muscle activities between soft and hard gum chewing (Wilcoxon signed‐rank test, paired *t*‐test) (Figure 2).

### 3.5 Phase

**FIGURE 3:**
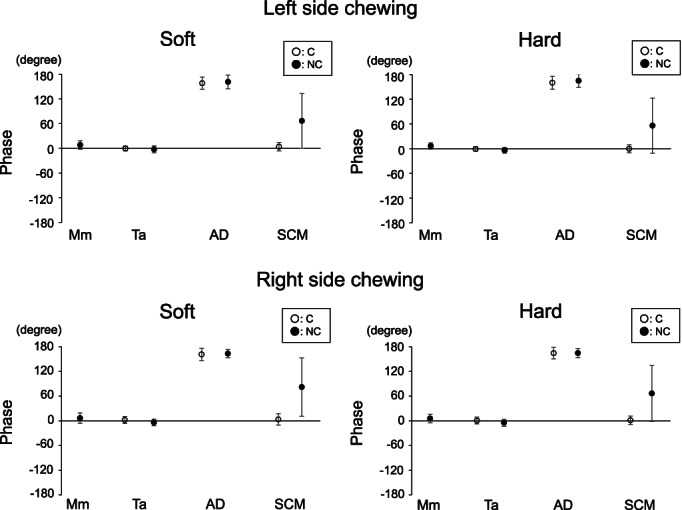
Phase for jaw and neck muscle activities. Mean and standard deviation of phase values for first peak frequency of jaw closing (NC‐mm, C‐/NC‐ta), jaw opening (C‐/NC‐AD), and neck (C‐/NC‐SCM) muscle activities are shown. No significant differences were seen for phase between soft and hard gum (Wilcoxon signed‐rank test, paired *t*‐test). The prefixes of ‘C’ (open circle) and ‘NC’ (black circle) denote the chewing side and non‐chewing side muscles, respectively. Mm, ta, AD, and SCM represent masseter muscle, anterior temporal muscle, anterior digastric muscle, and sternocleidomastoid muscle, respectively

Phase for the first peak frequency of jaw closing (NC‐Mm, C‐/NC‐Ta), jaw opening (C‐/NC‐AD), and neck (C‐/NC‐SCM) muscle activities was evaluated during right and left side chewing with soft and hard gum (Figure 3). Phase for jaw‐closing muscle and chewing side neck muscle activities was synchronized, while that for jaw opening muscle activity was presented as antiphase for jaw closing and chewing side neck muscle activities. Furthermore, phase for the non‐chewing side neck muscle was broadly distributed across jaw closing and jaw opening muscle activities. No significant difference was seen for phase of jaw closing (NC‐Mm, C‐/NC‐Ta), jaw opening (C‐/NC‐AD), and neck (C‐/NC‐SCM) muscle activities between soft and hard gum (Wilcoxon signed‐rank test, paired t‐test) (Figure 3).

### 3.6 Coherence

**FIGURE 4:**
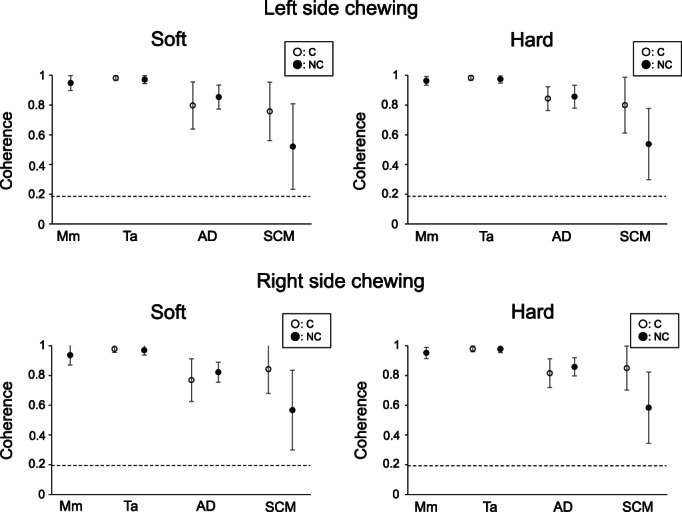
Coherence for jaw and neck muscle activities. Mean and standard deviation of coherence values for the first peak frequency of jaw closing (NC‐mm, C‐/NC‐ta), jaw opening (C‐/NC‐AD), and neck (C‐/NC‐SCM) muscle activities are shown. No significant differences were seen for coherence between soft and hard gum (Wilcoxon signed‐rank test, paired *t* test). The 95% confidence level (0.19) for the coherence spectrum is shown by a horizontal line. The prefixes of ‘C’ (open circle) and ‘NC’ (black circle) denote the chewing side and non‐chewing side muscles, respectively. Mm, ta, AD, and SCM represent masseter muscle, anterior temporal muscle, anterior digastric muscle, and sternocleidomastoid muscle, respectively

Coherence for the first peak frequency of jaw closing (NC‐Mm, C‐/NC‐Ta), jaw opening (C‐/NC‐AD), and neck (C‐/NC‐SCM) muscle activities was evaluated during right and left side chewing with soft and hard gum (Figure 4). No significant difference was seen for coherence of jaw closing (NC‐Mm, C‐/NC‐Ta), jaw opening (C‐/NC‐AD), and neck (C‐/NC‐SCM) muscle activities between soft and hard gum (Wilcoxon signed‐rank test, paired *t* test) (Figure 4).

## 4 DISCUSSION

The present subjects showed a common first peak frequency in power spectrum for all of the examined jaw and neck muscle activities. Nielsen et al. (1994) reported that the power spectrum of rectified leg muscle EMG activities during gait can be expressed as 1.0 Hz, with a large amplitude corresponding to stride‐cycle frequency. Furthermore, chewing frequency has been reported to range from 1.00–1.25 Hz (Ferreira et al., 2014; Rilo et al., 1998; Tartaglia et al., 2008; Youssef et al., 1997). Therefore, the first peak frequency noted in the present study may be interpreted as aggregation of rhythmicity and pattern generation of jaw and neck muscle activities during chewing performance.

Further, gain and phase in first peak frequency likely indicate the quantitative and temporal coordination of jaw and neck muscle activities during chewing performance, and coherence likely represents the functional coordination during jaw and neck muscle activities (Ishii et al., 2016). In addition, the present healthy subjects showed occlusal states comparable to those previously reported, including occlusal force (Narita et al., 2009; Sadamori et al., 2007), occlusal area, force AI, average pressure, and maximum pressure (Sadamori et al., 2007). Based on the associations between occlusal state and jaw and neck muscle activities previously reported (Hannam et al., 1977; Kibana et al., 2002; Ross et al., 2007; Tewksbury et al., 2018), the spectrum data obtained in this study may suggest the physiological features in jaw and neck muscle activities occur during chewing performance in healthy individuals.

### 4.1 Transfer function analysis for jaw and neck muscle activities during soft and hard gum chewing

Intermuscular EMG–EMG transfer function analysis for gain and phase parameters revealed the consistent quantitative and temporal coordination between jaw and neck muscle activities demanded by the chewing load using gum hardness in the healthy control subjects (Clark, 2015; Eyskens et al., 2015; Harms et al., 2019; Nakamura et al., 1999; Nakamura & Katakura, 1995), and which is considered to be predominantly generated by the circuits of “central rhythm and pattern generator” in the brainstem (Nakamura et al., 1999; Nakamura & Katakura, 1995; Takakusaki, 2017).

### 4.2 Coherence function analysis for jaw and neck muscle activities during soft and hard gum chewing

Intermuscular EMG–EMG coherence function analysis of chewing‐related jaw and neck muscle activities in this study may reveal the consistent functional coordination between jaw and neck muscle activities induced by chewing load using gum hardness (Ishii et al., 2016; Laine & Valero‐Cuevas, 2017). It has been reported that simultaneous activation of neck muscle activity during jaw clenching (Giannakopoulos, Hellmann, et al., 2013; Giannakopoulos, Schindler, et al., 2013; Kibana et al., 2002; Politti et al., 2010) and chewing performance (Guo et al., 2017; Häggman‐Henrikson & Eriksson, 2004; Häggman‐Henrikson et al., 2013; Igarashi et al., 2000; Shimazaki et al., 2006). In particularly, Igarashi et al. (Igarashi et al., 2000) noted the rhythmical neck muscle activity evoked by biting a wooden stick during cortically induced rhythmical chewing in rabbits, and also Häggman‐Henrikson et al. (2013) reported that sternocleidomastoid neck muscle was activated by hard gum chewing in healthy subjects.

### 4.3 Clinical implications

The intermuscular EMG–EMG transfer function and EMG–EMG coherence function analyzes presented the consistent functional coordination in jaw and neck muscle activities induced by chewing load using gum hardness in healthy subjects. These physiological characteristics in healthy subjects may be clinically utilized to quantitatively evaluate the pathophysiological features in TMD and/or whiplash disorders (Grönqvist et al., 2008; Haketa et al., 2006; Kurita et al., 2001; Lampa et al., 2017; Silveira et al., 2014) from the viewpoints of the consistent functional coordination of jaw and neck muscle activities induced under the condition of chewing load using gum hardness.

## 5 CONCLUSIONS

This study was conducted to quantitatively clarify the effects of hardness of gum on the coordinated features in jaw and neck muscle activities in healthy individuals, using intermuscular EMG–EMG transfer and EMG–EMG coherence function analyzes. Chewing load using gum hardness presented the consistent chewing rhythm, the temporal and power coordination, and the functional coordination in jaw and neck muscle activities, accompanying increased jaw and neck muscle activities in healthy subjects. It is concluded that jaw and neck muscles coordination is maintained under the condition of the chewing load using gum hardness in healthy individuals.

## CONFLICT OF INTEREST

Takeshi Uchida and Ikuo Kantake are employed by Dental Support Co. Ltd. Neither has any potential conflicts of interest to declare with respect to research, authorship, and/or publication of this article. The remaining authors declare that the research was conducted in the absence of any commercial or financial relationships that could be construed as a potential conflict of interest.

## Data Availability

The data that support the findings of this study are available from the corresponding author upon reasonable request.
